# Screening of Nonpathogenic *Fusarium* Species Suppressing Fusarium Crown and Root Rot in Asparagus Using Wheat Bran as an Inoculum Carrier

**DOI:** 10.1264/jsme2.ME25063

**Published:** 2026-05-20

**Authors:** Yosuke Maeda, Seishi Akino, Norio Kondo

**Affiliations:** 1 Graduate School of Agriculture, Hokkaido University, Kita-ku Kita 9 Nishi 9, Sapporo, 060–8589, Japan; 2 Research Faculty of Agriculture, Hokkaido University, Kita-ku Kita 9 Nishi 9, Sapporo, 060–8589, Japan; 3 The Hokkaido University Museum, Kita-ku Kita 10 Nishi 8, Sapporo, 060–0810, Japan

**Keywords:** *Fusarium oxysporum*, asparagus, biological control, competition

## Abstract

Fusarium crown and root rot (FCRR), caused by the fungus *Fusarium oxysporum* f. sp. *asparagi*, is one of the most destructive diseases affecting asparagus worldwide. Despite evidence of the suppression of FCRR by several nonpathogenic *F. oxysporum* *sensu lato* strains in laboratory experiments, biological control trials in FCRR-infested fields have not consistently demonstrated the effectiveness of these strains. Therefore, we screened nonpathogenic *Fusarium* spp. strains for the biological control of FCRR with the modified inoculation method considering the colonization to soil of biocontrol agents (BCAs). Two strains from different lineages (SM007 and FAoc5), inoculated by irrigating a bud-cell suspension and applying cultured wheat bran medium around the root of the transplants, effectively suppressed FCRR on young asparagus seedlings. These strains also suppressed FCRR on mature second-year plants to some extent by the application of cultured wheat bran medium as an inoculation carrier. Both strains exhibited the capacity to impede chlamydospore germination of the pathogen when glucose was incorporated into soil. Although SM007 sometimes failed to show biocontrol effects, differences in efficiency appeared to be related to the fungal density colonizing wheat bran medium. These results suggest that the screened strains interact with the pathogen and also that the amount of BCAs introduced into soil is closely related to their biocontrol efficacy.

Asparagus (*Asparagus officinalis* L.) is an Asparagaceous perennial crop grown worldwide. A significant constraint on asparagus production is the syndrome of ‘asparagus decline’. Over time, crowns yield smaller and fewer spears and eventually die (Elmer, 2004, 2015). An increase in the density of soil pathogens, such as *Fusarium oxysporum* f. sp. *asparagi* (Foa) and *Fusarium proliferatum* (Fp), causing Fusarium crown and root rot (FCRR), is considered to cause that decline (Reid *et al.*, 2002; Doumen *et al.*, 2018).

Nonpathogenic strains of *Fusarium* species, mainly belonging to the *F. oxysporum* (Fo) species complex (SC), are well-known biocontrol agents (BCAs). A number of strains, such as Fo47 (Alabouvette *et al.*, 1993) and CS-20 (Larkin and Fravel, 1998), have been intensively exami­ned and found to be effective against various crop diseases caused by *Fusarium* species. Some strains were shown to efficiently suppress disease in asparagus under controlled environments (Block *et al.*, 1997; Damicone and Manning, 1982; Tu *et al.*, 1990; He *et al.*, 2002; Reid *et al.*, 2002; Elmer, 2004; He and Wolyn, 2005; Liu and Matsubara, 2016). However, the findings of field experiments have been inconsistent, indicating the need for further research on the performance and persistence of these BCAs under field conditions (Block *et al.*, 1997; Elmer, 2004).

The efficiency of BCAs is often assessed based on their ability to colonize host plants or soil (Mandeel and Baker, 1991; Shishido *et al.*, 2005). Observations of tomato roots revealed that while pathogenic strains colonized vascular tissues, nonpathogenic strains of Fo primarily remained on the root surface, rarely reaching vascular tissues (Olivain and Alabouvette, 1997, 1999; Olivain *et al.*, 2006). Similarly, studies on *Arabidopsis* showed that the nonpathogenic strain Fo47 was rarely detected in vascular tissues, except in lateral roots and the elongation zone (Martínez-Soto *et al.*, 2023). These findings suggest that the persistence of nonpathogenic Fo in the soil, rather than within plant roots, is crucial for its establishment.

Yang *et al.* (2011) reported that *Trichoderma harzianum* SQR-T037 colonized the cucumber rhizosphere and bulk soil more effectively when applied with a fermented organic fertilizer than when administered as a conidial suspension, leading to stronger disease suppression against Fo. Moreover, experiments using wheat bran as an inoculum source revealed that *T. viride* inoculated on bran as mycelia remained in the soil at higher densities than when inoculated as conidia (Lewis and Papavizas, 1984). These findings indicate that the application of organic materials, such as wheat bran inoculated with mycelia, has potential as a reliable method for maintaining high BCA densities in the soil.

In the present study, we aimed to identify nonpathogenic *Fusarium* species belonging to Fo SC (hereafter referred to as Fo) strains that suppress FCRR using wheat bran as an inoculum carrier. We also investigated the direct effects on the pathogen and phylogeny of the screened strains.

## Materials and Methods

### Inoculum preparation

The pathogenic Foa strain R2-5, originally from an asparagus cultivated in Hokkaido, was used to prepare infested soil. The pathogenic strain was cultured in green pea broth (GPB, Tanaka *et al.*, 2010) on a reciprocating shaker (120 rpm) at room temperature for 4–7 days. After culturing, the medium was filtered using ADVANTEC No. 1 filter paper, and mycelia containing bud-cells were homogenized using a Waring blender (CM-100; As One). The homogenized mycelial suspension was incorporated into the potting mix (Pot Ace N; Katakura & Co-op Agri), which was then air-dried for 2 weeks to allow chlamydospore formation. Soil pathogen density was adjusted to 1.0×10^4^ cfu g^–1^ soil.

To prepare bud-cell suspensions of candidate BCA strains, strains suppressing FCRR via a root-dip inoculation were selected based on greenhouse experiments (Table 1 and S1). Each strain was cultured in GPB on a reciprocating shaker for 4–10 days. The cultured medium was then filtered through three layers of gauze to remove mycelia and pelleted by centrifugation at 3,000 rpm for 10 min. The resulting bud-cell pellets were resuspended in sterilized distilled water (SDW), and their density was assessed using a hemocytometer.

To prepare wheat bran medium, two 8-mm mycelial plugs of each nonpathogenic strain cultured in green pea agar (GPA) for 7 days were mixed into autoclaved wheat bran medium (121°C for 60 min). This medium consisted of wheat bran, vermiculite, and tap water in a 1:1:3 (w/w) ratio and was incubated at 24°C for two weeks.

### Screening of Fo strains that suppress FCRR inoculated via wheat bran medium and the bud-cell inoculum

A greenhouse experiment was conducted to evaluate potentially effective strains using an inoculation method for the optimized field application of BCAs. Asparagus seeds (cv. Windel) were surface-sterilized with 1% sodium hypochlorite for 60 min, rinsed three times with SDW, and grown in a greenhouse (located in Sapporo, Hokkaido, Japan) for three weeks in a mixture of vermiculite-mixed potting mix (1:4, [v/v]). To apply the treatments, 50 mL of the bud-cell suspension (1.0×10^6^ bud-cells mL^–1^) from each nonpathogenic strain was irrigated into pots containing 10 asparagus seedlings, followed by an additional week of incubation. Five seedlings were then transplanted into 1.8-L pots containing infested soil and 10 g of wheat bran medium inoculated with each strain was applied around the root of the transplants.

As the negative control (NC), seedlings irrigated with SDW were transplanted into non-infested soil containing autoclaved non-inoculated bran (NIB). As the positive control (PC), seedlings irrigated with SDW were transplanted into infested soil containing NIB. Plants were grown in the greenhouse (minimum temperature of 15°C or higher, February 7 to March 14, 2023) for 5 weeks and the number of plants exhibiting symptoms such as yellowing or wilt was recorded. Disease incidence at each time point (days post inoculation; dpi) was calculated based on the number of plants showing chlorosis or wilt symptoms relative to the total number of plants per plot.

The area under the disease-progress curve (AUDPC) for each treatment was calculated using the *audpc* function from the *agricolae* package in R (version 4.4.2). AUDPC values (excluding NC) were statistically compared to PC using Dunnett’s test. Treatments were arranged in a randomized block design with four replicate pots per treatment, and the experiment was conducted once.

### Mature plant experiments

The efficiency of BCAs at suppressing FCRR in mature asparagus plants was tested. A 1:4 (v/v) mixture of vermiculite and field soil collected from Hokkaido University’s experimental field (in Sapporo, Hokkaido, Japan) was autoclaved at 121°C for 20 min on 2 consecutive days. Asparagus seeds (cv. Mary Washington) were sown in 1/5,000a Wagner pots containing potting mix on 10 May 2022 and cultivated in the Hokkaido University field for 7 months. On 1 December 2022, aboveground tissues were removed, and pots containing roots were buried under snow throughout the winter. After a 1-year cultivation period, most seedlings developed root lesions. The pathogen was isolated from these lesions with Komada medium (Komada, 1975).

The nonpathogenic strains SM007 and FAoc5 were inoculated onto wheat bran medium. Each 1-year-old asparagus root was trimmed, leaving the bottom 5 cm of the crown, and was then transplanted into Wagner pots containing infested field soil. At the time of transplantation, 10 g of wheat bran medium and a chemical fertilizer (8:8:8, N:P:K) were applied around the root. Two control treatments were established: seedlings transplanted into non-infested autoclaved field soil with NIB (NC), and seedlings transplanted into infested field soil with NIB (PC). Treatments were arranged in a randomized block design with three plots, each containing 10 plants per treatment.

Plants were grown for 165 days, from 23 May to 3 November 2023. Disease incidence was monitored until almost all plants were‍ ‍diseased (121 dpi). Disease severity from 97 to 121 dpi was‍ ‍assessed using the disease index. The aboveground disease index was scored on a scale of 0 to 4, where 0=no symptoms, 1=chlorosis or wilt on less than 33%, 2=chlorosis or wilt on 34–66%, 3=chlorosis or wilt more than 67%, and 4=death. Disease severity was calculated as Σ ([1A+2B+3C+4D]/4N)×100, where A, B, C, and D represent the number of plants rated 1, 2, 3, or 4, respectively, and N is the total number of plants. To assess the impact of the pathogen on plant biomass production, aboveground tissues were removed at 121 dpi, and emerging spears were grown for an additional 4 weeks. Aboveground tissues that formed within one month were harvested, and dry weight was measured for each plot. Following stem harvesting, plants remained in the field for another month (until 3 November), but no further spears emerged, signaling the end of the cultivation period. Root tissues were then harvested to evaluate disease impact. The number of plants displaying lesions on more than 67% of their roots in each plot was recorded, and root dry weight was measured after assessing disease severity. When measuring plant biomass, the aboveground parts were weighed, excluding individuals that had not yet produced stems prior to the disease evaluation, whereas root parts were weighed in all plants. Therefore, both datasets represent the weight per plant in each experimental plot. A logistic regression anal­ysis of the number of severely diseased plant roots was performed using a generalized linear model (GLM) with the *glm* function in R.

### Evaluation of effects of wheat bran medium application

Greenhouse experiments were conducted to confirm the effects of the sole application of wheat bran medium. The inoculated wheat bran medium was produced following the established method, and a portion was subjected to a serial dilution procedure to assess the density of SM007 cfu g^–1^ fresh bran.

To confirm that NIB did not enhance the activity of the pathogen, re-autoclaved wheat bran medium following the inoculation of SM007 (inoculated autoclaved bran, IAB) was incorporated into the experiment. Four-week-old asparagus seedlings (cv. Mary Washington) were grown using the same method as that for screening. Following the treatment, seedlings were transplanted into infested soil and grown for four weeks. Four treatments were prepared: the first group consisted of plants transplanted into non-infested soil treated with IAB, serving as the Mock treatment. The second group involved plants transplanted into infested soil with NIB, representing the NIB treatment. The third group involved plants transplanted into infested soil with IAB, representing the IAB treatment. The fourth group consisted of plants transplanted into infested soil with SM007-inoculated bran, serving as the SM007 treatment.

Aboveground disease incidence was assessed using previously described methods. Treatments were arranged in a randomized block design with four replicate pots per treatment, and the experiment was conducted twice (transplanting dates: September 27 and October 11, 2023). Given the ten-fold difference in SM007 density between the two experiments, AUDPC values for each treatment were analyzed separately.

### Effects of nonpathogenic strains of Fo on chlamydospore germination of Foa

In the greenhouse experiments described above, the nonpathogenic strains inoculated onto organic matter were assumed to affect the Foa pathogen directly. We assessed their ability to inhibit chlamydospore germination in Foa. The experiment followed the methodology outlined in a previous study (Widodo, 2000). Infested soil containing chlamydospores of pathogenic strain R2-5 was prepared using a previously described method with a sterilized potting mix, and fungal density was adjusted to 1.0×10^3^ cfu g^–1^ soil. The soil was stored at 4°C until used. To investigate the effects of soil nutrients, different concentrations of glucose-asparagine (5:1) solution (0, 0.2 or 0.4 mg g^–1^ soil) were added to a vial containing 1 g of infested soil. One-milliliter bud-cell suspensions (1.0×10^6^ bud-cells mL^–1^) of either nonpathogenic strain SM007 or FAoc5 were added to each vial. Soil moisture was adjusted to 25% with SDW, and treated soil was incubated at 24°C for 22 h. After the incubation, soil samples were stained with lactophenol cotton blue. To extract chlamydospores for observations under a microscope, 1 ml SDW was added to each sample, and the bottles were shaken to release the spores into the supernatant. The supernatant was placed onto glass slides for examination. One hundred chlamydospores were observed for each treatment, and their germination rate was calculated. Experiments were conducted three times, with two replicates per trial. The effects of glucose concentrations, the strain inoculation, and their interactions were analyzed using GLM.

### Phylogenetic anal­ysis of strains used in this study

DNA from the strains used in this study was extracted using FastDNAkit (MP Biomedicals). The partial nucleotide sequences of two gene regions (translation elongation factor 1-α: *EF-1α* and RNA polymerase second largest subunit: *RPB2*, O’Donnell *et al.*, 2022) were amplified using PCR. The primers and conditions of amplification are listed in Table S2. Sequence data from the ex-type strains of Fo SC (shown in Table S3) were retrieved from the National Center for Biotechnology Information (NCBI). The sequences obtained were aligned independently and concatenated. Phylogenetic trees were generated by the Maximum Likelihood (ML) method using MEGA 11 (Tamura *et al.*, 2021).

## Results

### Screening of Fo strains that suppress FCRR inoculated via wheat bran medium and the bud-cell inoculum

**Fig. 1.**
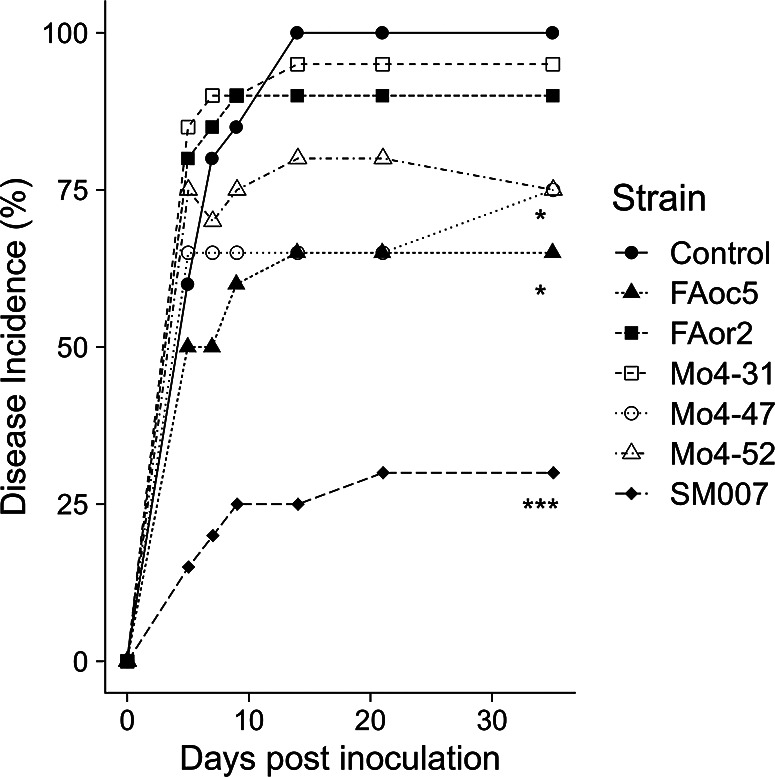
Disease incidence of Fusarium crown and root rot in asparagus seedlings after transplanting them into pathogen-infested soil. Each treatment involved the application of a nonpathogenic strain through bud-cell suspension irrigation and/or inoculated wheat bran medium incorporation. Each plot represents the mean value of each treatment at each dpi, except for NC. * and *** indicate significant differences (*P*<0.05 and *P*<0.001, respectively) from PC, based on Dunnett’s test for area under the disease-progress curve values.

Plants in all treatments, except NC, exhibited some degree of wilt symptoms. At 35 dpi, when all plants in PC had died, the inoculation of SM007 or FAoc5 significantly reduced disease incidence to 30% (*P*<0.001) and 65% (*P*<0.05), respectively (Fig. 1). Although Mo4-47 also reduced the AUDPC value, disease incidence was high in the early dpi. Based on these results, SM007 and FAoc5 were selected for further experiments. No significant differences were observed in treatments inoculated with other strains. However, in these treatments, plants initially showed rapid disease progression in the early dpi period, followed by a slower rate of disease development.

### Mature plant experiments

The pathogen isolated from lesions that appeared during the seedling cultivation was identified as Fp via sequencing of the *EF-1α* region (accession number LC872907). Consequently, disease symptoms were also observed in NC plants. Disease incidence and severity were evaluated based on overall wilt or root lesions because it was not possible to differentiate symptoms between Fp and Foa. Additionally, some plants died between transplanting and shoot emergence in both NC and other treatments. These plants were excluded from the disease severity evaluation because they were more likely to be affected by Fp than by Fo.

**Fig. 2.**
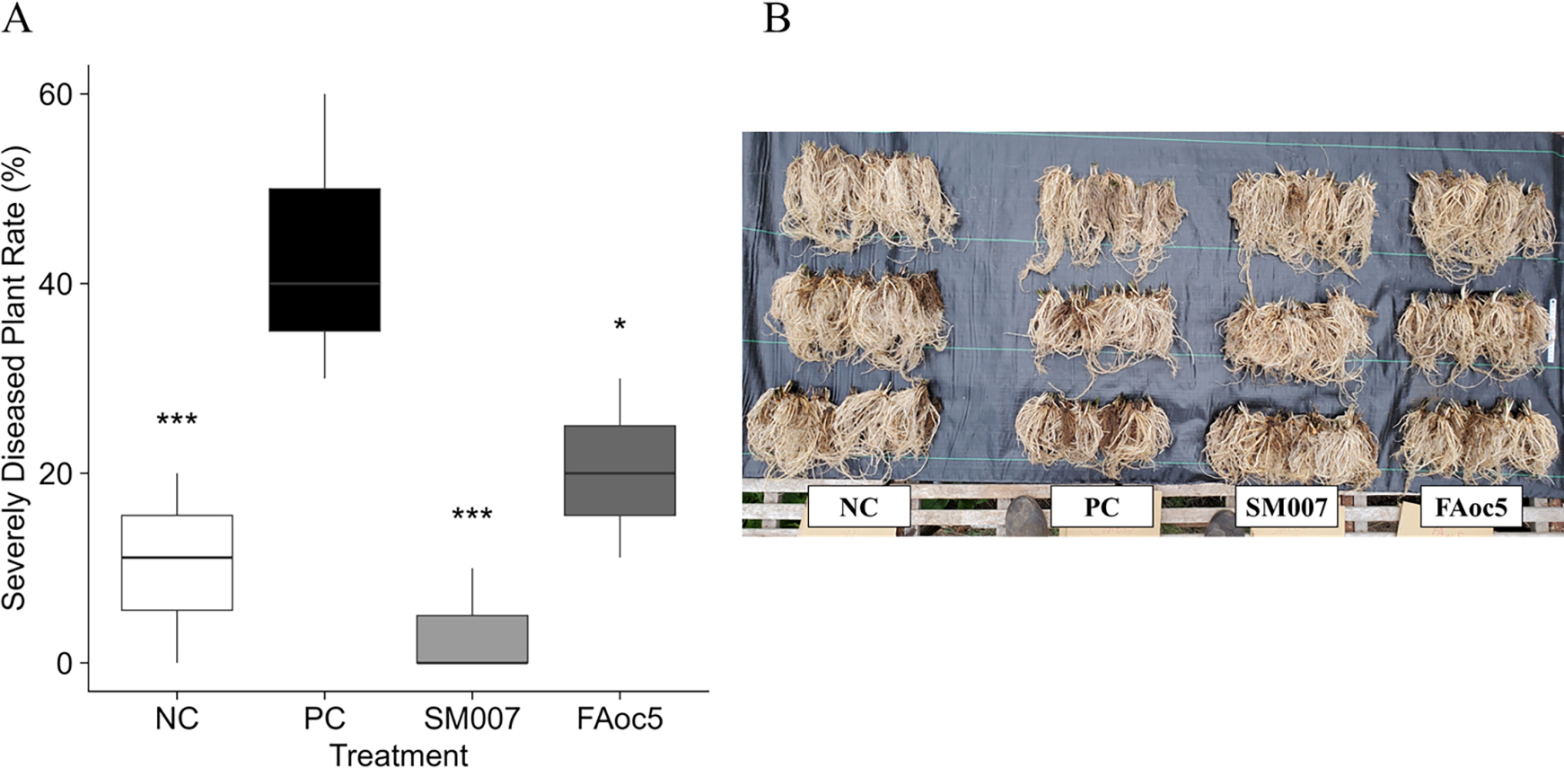
Mature seedling experiments. (A) Ratio of plants exhibiting root lesions affecting more than 67% of the root system and (B) visible appearance of plant roots after cultivating mature plants in the Hokkaido University field following transplantation into pathogen-infested field soil and the incorporation of wheat bran medium inoculated with each nonpathogenic strain. Brown lesions represent a symptom of Fusarium crown and root rot. * and ** indicate significant effects (*P*<0.10 and *P*<0.01, respectively) on the number of severely diseased plants, as assessed by a logistic regression anal­ysis. NC and PC represent negative and positive controls, respectively.

Disease development was generally slower in treatments with nonpathogenic strains, particularly with SM007; however, no significant suppressive effect on disease progression in aboveground tissues was observed relative to PC (Fig. S1). Furthermore, aboveground disease severity on each dpi was significantly lower for each strain than for PC (Table 3). Root disease severity after 165 days of cultivation was also examined to assess the percentage of severely diseased plants. Significant effects were detected in comparisons of PC with all other treatments in terms of root disease development (Fig. 2). While NC plants still exhibited root lesions, the percentage of severely diseased plants was significantly lower than in PC plants (*P*<0.01). Treatments involving wheat bran medium inoculated with SM007 or FAoc5 also reduced the rate of severely diseased plants (*P*<0.01 and *P*=0.067, respectively), although the extent of disease suppression varied between strains. A similar result was obtained for asparagus biomass production. Although Tukey-Kramer’s test did not yield significant differences in either the stem or root biomass, the dry weight of both plant organs in NC plants and inoculations with nonpathogenic strains was higher than in PC plants in all experimental plots (Table 2).

### Evaluation of effects of wheat bran medium application

**Fig. 3.**
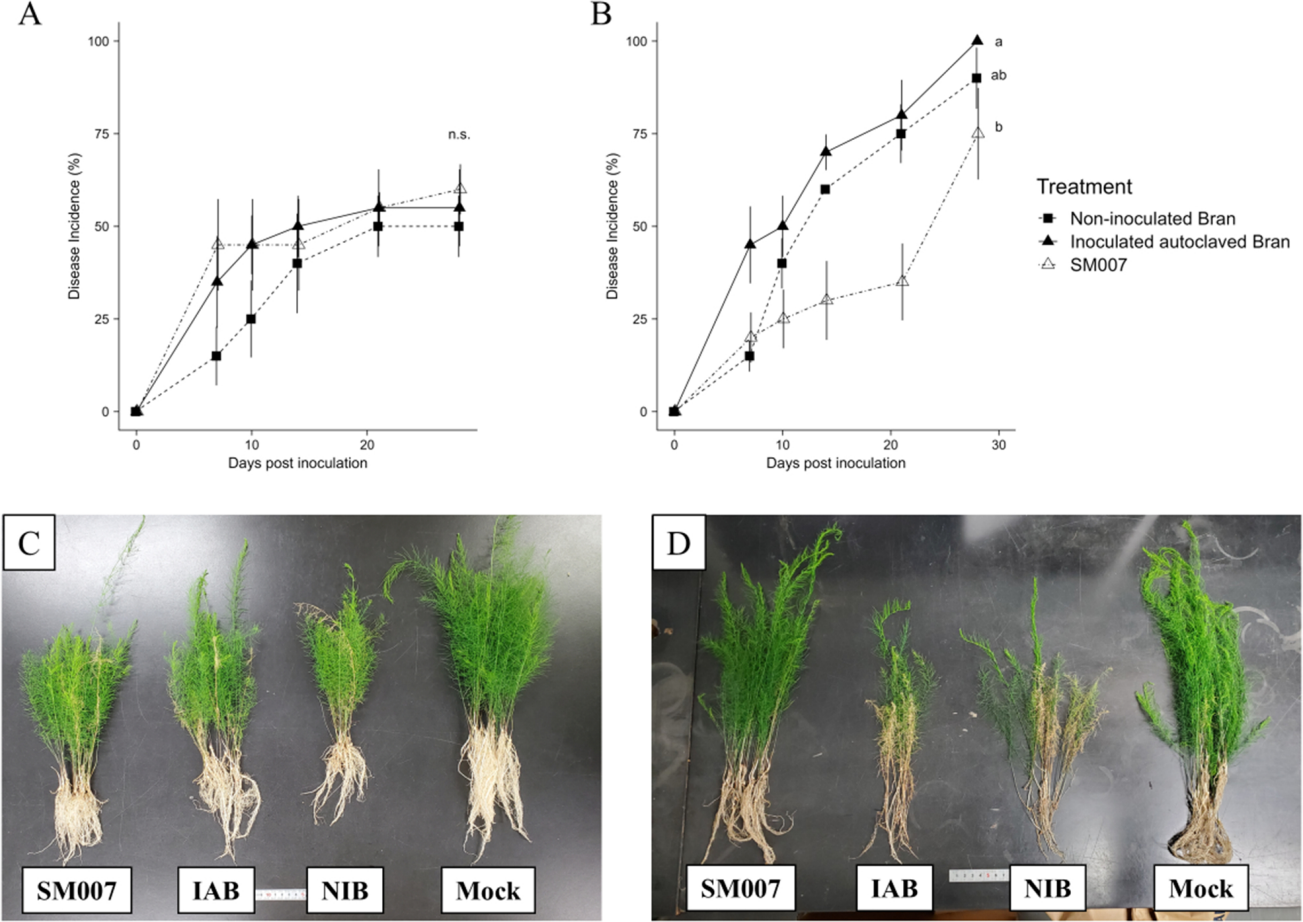
Disease incidence and the symptoms of Fusarium crown and root rot after the transplantation of asparagus seedlings into pathogen-infested soil following the application of treated wheat bran medium (10 g per pot). Each plot represents the mean disease incidence for each treatment at each dpi. Results from two experiments are shown separately due to differences in the density of nonpathogenic strain SM007 colonizing wheat bran medium. (A) and (C) First experiment (5.4×10^4^ cfu g^–1^ fresh bran); (B) and (D) Second experiment (8.2×10^5^ cfu g^–1^ fresh bran). Different letters indicate significant differences (*P*<0.05) based on Tukey-Kramer’s test for area under the disease-progress curve values (*n*=4). “n.s.” means no significant difference based on Tukey-Kramer’s test (*P*>0.05).

The two experiments showed clear differences in both the density of SM007 colonized in wheat bran medium and its suppressive effects. SM007 colonization densities in wheat bran medium were 5.4×10^4^ cfu g^–1^ bran in the first experiment and 8.2×10^5^ cfu g^–1^ bran in the second. In the first experiment, despite the low disease levels observed in PC, no suppressive effect of SM007 inoculated with wheat bran medium was detected (Fig. 3A and C). However, in the second experiment, severe wilt symptoms developed, and the suppression of disease progression by SM007 was significantly stronger than that by IAB. Although the difference between SM007 and NIB was not significant, disease incidence with SM007-inoculated wheat bran medium remained markedly lower during early dpi (Fig. 3B and D). In consideration of the severity of symptoms caused by the pathogen, disease progression was significantly slower with SM007 than with both infested treatments, as evaluated using AUDPC values based on disease severity (Fig. S2). Additionally, no significant differences were observed between NIB and IAB in either experiment.

### Effects of each nonpathogenic strain on chlamydospore germination of Foa

**Fig. 4.**
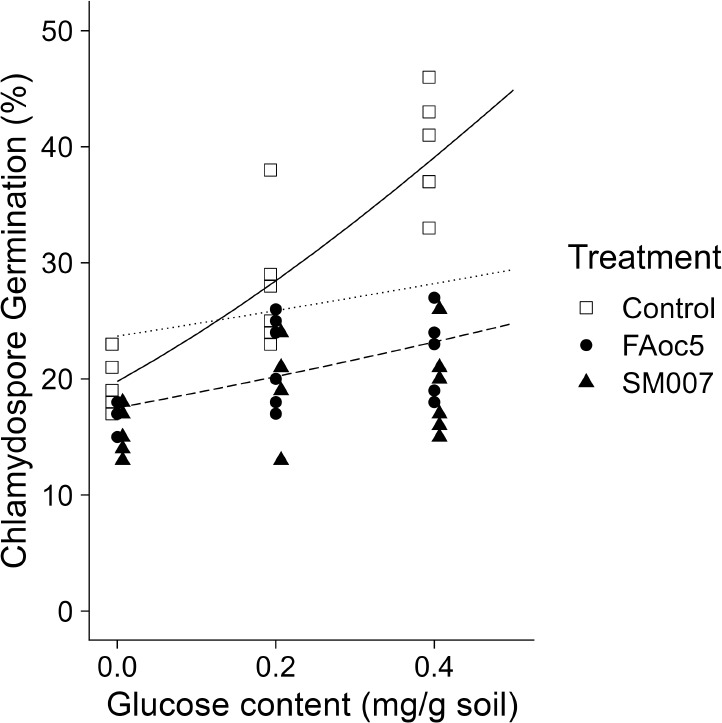
Chlamydospore germination rate of the pathogen (1.0×10^3^ chlamydospores g^–1^ soil) treated with 1 ml of the bud-cell suspension of nonpathogenic strains (1.0×10^6^ bud-cells mL^–1^) at varying glucose levels. Each plot represents the values of individual replicates. Lines represent the relationship equations created based on the coefficients obtained from the logistic regression: solid line for Control, dotted line for FAoc5, and dashed line for SM007. Significant interactions between the strain treatment and glucose content (*P*<0.01) were detected based on a logistic regression anal­ysis.

The logistic regression anal­ysis indicated that the model incorporating the effects of nonpathogenic strains, glucose content, and their interactions produced the lowest Akaike information criterion value. Both of the nonpathogenic strains significantly reduced pathogen germination rates at each soil glucose level. In NC, germination rates increased from 20.2 to 39.5% as glucose levels increased. However, germination rates in soil treated with the nonpathogenic strains SM007 and FAoc5 remained at 19.1 and 22.5%, respectively, even at a glucose concentration of 0.4 mg g^–1^ soil. While the reduction in germination rates without glucose supplementation was limited to 3.3–4.5%, the difference between the control and each treatment increased to 20.3 and 17%, respectively, under glucose-enriched conditions (Fig. 4).

### Phylogenetic anal­ysis of strains used in this study

**Fig. 5.**
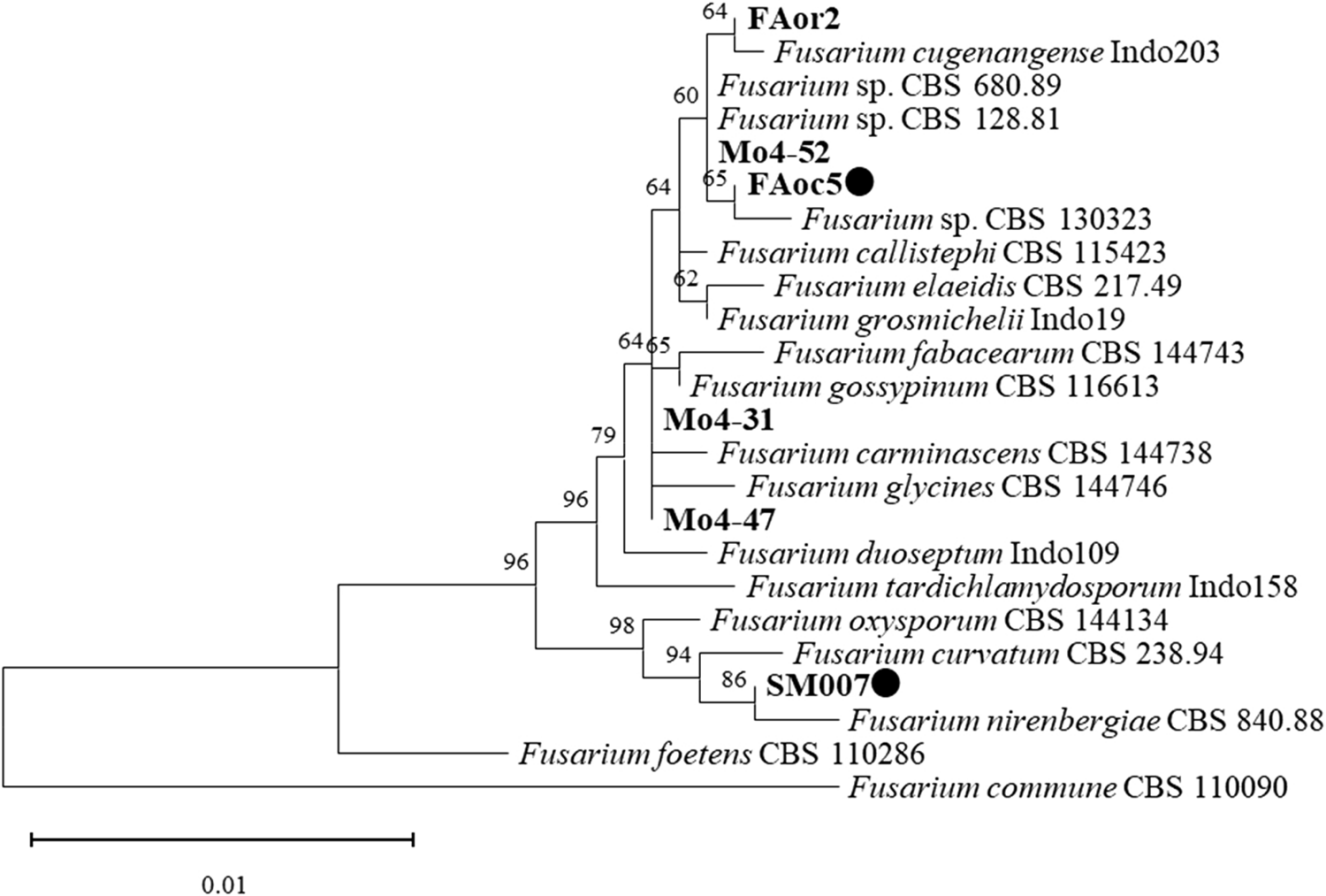
Maximum likelihood bootstrapped phylogenies inferred from partial *EF-1α* (524 bp) and *RPB2* (859 bp) of 5 tested strains and relative species. Two strains screened in this study belong to different clades. Numbers by nodes represent ML-BS support based on 500 pseudoreplicates of the data. *Fusarium commune* CBS 110090 was used as an outgroup. *Fusarium vaughaniae* was excluded from the construction of the phylogenetic tree because *RPB2* sequences from the ex-type strain were not registered in NCBI.

A phylogenetic anal­ysis of strains used in this study was conducted using two gene regions (*EF-1α* and *RPB2*). The results obtained showed that SM007 and FAoc5 were not grouped into a certain clade (Fig. 5). Furthermore, SM007 was closely related to *Fusarium nirenbergiae* L. Lombard et Crous, whereas FAoc5 was close to *Fusarium vaughaniae* Y. P. Tan & Conroy, based on homology searches using Fusarioid ID (Crous *et al.*, 2021).

## Discussion

In the present study, we identified two nonpathogenic strains SM007 and FAoc5, that suppressed FCRR development even in mature plants. SM007, originally isolated from the soil of onion fields, was shown to be an effective BCA against Fusarium basal rot and Botrytis leaf blight (Widodo, 2000; Yamamoto *et al.*, 2007; Nogawa and Kondo, 2020). On the other hand, FAoc5 was isolated from the crowns of asparagus grown in field soil at Hokkaido University.

The results of the phylogenetic anal­ysis of strains used in this study showed that these strains were not grouped within a single clade and were classified as distinct species according to the classification system proposed by Lombard *et al.* (2019). Constantin *et al.* (2021) reported that the co-inoculation of nonpathogenic Fo strains with a pathogenic strain at a 1:1 ratio using the root-dipping method resulted in most strains exerting disease suppressive effects. They also noted that the phylogenetic relationships among these strains were inconsistent. Although the biocontrol effects of these strains were mentioned as the induction of plant resistance in their study, the findings obtained are consistent with the present results in that biocontrol ability was not confined to a single lineage.

In mature plant experiments, seedlings exhibited root lesions caused by Fp before transplanting. Root lesions were also observed in NC plants after transplanting, indicating that Fp caused damage prior to their exposure to Fo. These results suggest that the BCAs used in this study successfully suppressed disease caused by Fo, even when plants had previously been infected by another pathogen before the BCA inoculation (Fig. 2 and Table 2). However, it remains unclear whether these BCAs directly control Fp. In practical field cultivation, BCAs must be effective against multiple pathogens, which suggests that further research is needed on their broader applicability.

In the screening experiment, the combination of wheat bran medium application and bud-cell irrigation was effective as an inoculation method for the screened strains (Fig. 1). Therefore, we confirmed the contribution of the wheat bran medium treatment to disease control in additional experiments. While we did not observe a biocontrol effect in the first experiment, significant disease suppression was observed in the second experiment (Fig. 3 and S2). Furthermore, in additional experiments to investigate the effects of bud-cell irrigation, while irrigation alone or the combination of irrigation and wheat bran medium addition slightly suppressed disease incidence at an early dpi, wheat bran medium application exerted neither a biocontrol effect as a sole treatment nor an additive effect on bud-cell irrigation (Fig S3). In this experiment, although we did not quantify the density of SM007 colonizing wheat bran medium, a shortage of the fungus was presumed. Therefore, these results suggest the lack of a synergistic effect between these two application methods and also that the density of the introduced BCAs is crucial for biocontrol potential. The 15-fold difference in fungal density on wheat bran medium associated with biocontrol effects supports this interpretation. Furthermore, a previous study reported that the plant growth-promoting effect of SM007 was dependent on its fungal density (Kioka, Y., Noguchi, K., and Kondo, N. Materials for promoting plant growth and controlling disease and cultivating soil for raising seedlings containing the materials [JP2004307342A], 1 April 2003, Japan Patent Office.). The effectiveness of application methods and BCAs is not simply a matter of whether they work; it will be important to elucidate the conditions under which they function.

Furthermore, SM007 and FAoc5 may exert direct effects on the pathogen, such as nutrient competition. Consistent with previous studies (Mandeel and Baker, 1991; Larkin and Fravel, 1999; Widodo, 2000; Iida *et al.*, 2022), we confirmed competitive interactions between BCAs and the pathogen by examining spore germination by the pathogen. In this experiment, we observed the significant inhibition of chlamydospore germination by SM007 and FAoc5 when applying glucose (Fig. 4), whereas the cell wall degradation of ungerminated chlamydospores was not detected, indicating that competition for carbon sources was more essential than antibiosis. In addition, because we used NIB as PC in the screening, the effects of NIB as a carbon source on the pathogen were also evaluated by comparing NIB to IAB. There were no significant differences between NIB and IAB in both experiments, indicating that NIB was valid to use as PC (Fig. 3).

Several factors may contribute to the difference observed in control efficiency among individual strains, including variations in metabolites, host interactions, colonizing speed, colonization density, and inoculum sources. The required density for disease suppression varied among prominent strains, such as Fo47 and CS-20, which are effective against multiple crops. Fo47 primarily competes with the pathogen rather than inducing plant resistance and requires a density of at least 10^4^ chlamydospores g^–1^ soil, regardless of the pathogen inoculum density. In contrast, CS-20, which exhibits a weak ability for nutrient competition and induces plant resistance, requires only 10^2^ chlamydospores g^–1^ soil to be effective, even when the pathogen density reaches 10^5^ chlamydospores g^–1^ soil (Larkin and Fravel, 1999). These differences highlight the importance of strain-specific mechanisms in disease suppression and emphasize that the unique characteristics of each strain play a crucial role in biocontrol efficacy. Although nutritional competition between BCAs and the pathogen was suggested in the present study, further investigations are necessary to clarify the involvement of other mechanisms, such as the induction of plant resistance and antibiosis. To achieve more sustainable disease management, further studies need to investigate their colonization dynamics in plants and soil as well as ecological and metabolic differences among strains in order to optimize their use and integration into asparagus production systems.

## Citation

Maeda, Y., Akino, S., and Kondo, N. (2026) Screening of Nonpathogenic *Fusarium* Species Suppressing Fusarium Crown and Root Rot in Asparagus Using Wheat Bran as an Inoculum Carrier. *Microbes Environ ***41**: ME25063.

https://doi.org/10.1264/jsme2.ME25063

## Supplementary Material

Supplementary Material

## Figures and Tables

**Table 1.** Names and origins of strains used as biocontrol agents.

Strain	Origin	*EF-1α* ^a^	*RPB2* ^b^
SM007	Onion field soil	LC904126	LC904132
FAoc5	Crown of asparagus	LC904125	LC904128
Mo4-31	Onion field soil	LC904123	LC904130
Mo4-52	Onion field soil	LC904127	LC904133
FAor2	Root of asparagus	LC904122	LC904129
Mo4-47	Onion field soil	LC904124	LC904131

^a, b^ Accession numbers of each gene region in DDBJ/EMBL/GenBank databases.

**Table 2.** Shoot and root dry weights of asparagus.

	Shoot Dry Weight^a^ (g plant^–1^)	Root Dry Weight^b^ (g plant^–1^)
Negative Control	10.8±1.31	13.1±2.96
Positive Control	7.77±0.21	8.13±1.25
SM007	10.2±1.37	13.9±3.54
FAoc5	8.49±0.52	10.6±2.26

^a, b^ Values in each treatment represent means and standard deviations (*n*=3). There were no significant differences based on the results of Tukey-Kramer’s test (*P*>0.05).

**Table 3.** Shoot disease severity after transplanting mature plants into pots containing pathogen-infested soil.

	Disease severity (%)							
	Days post inoculation							
	97		106		113		121	
Negative Control	27.5±2.04	a	29.26±4.21	a	29.17±5.89	a	29.17±5.89	a
Positive Control	47.5±2.04	b	43.33±1.18	b	40.83±1.18	b	40±0	b
SM007	28.3±5.14	a	26.67±5.14	a	26.67±6.24	a	26.67±4.25	a
FAoc5	32.5±6.12	a	29.17±5.89	a	27.5±5.40	a	27.5±3.54	a

Values in each treatment represent means and standard deviations (*n*=3). Different letters indicate significant differences (*P*<0.05) based on the Steel Dwass test.
