# Supplementary material for: Screening of Nonpathogenic Fusarium Species Suppressing Fusarium Crown and Root Rot in Asparagus Using Wheat Bran as an Inoculum Carrier
Source: Microbes Environ. 2026 May 20;41(2):ME25063. doi: 10.1264/jsme2.ME25063 (PMC13293699; doi:10.1264/jsme2.ME25063)
Supplement: Supplementary file 1 — Supplementary Material [file 41_25063_s1.pdf]

Supplementary Figures

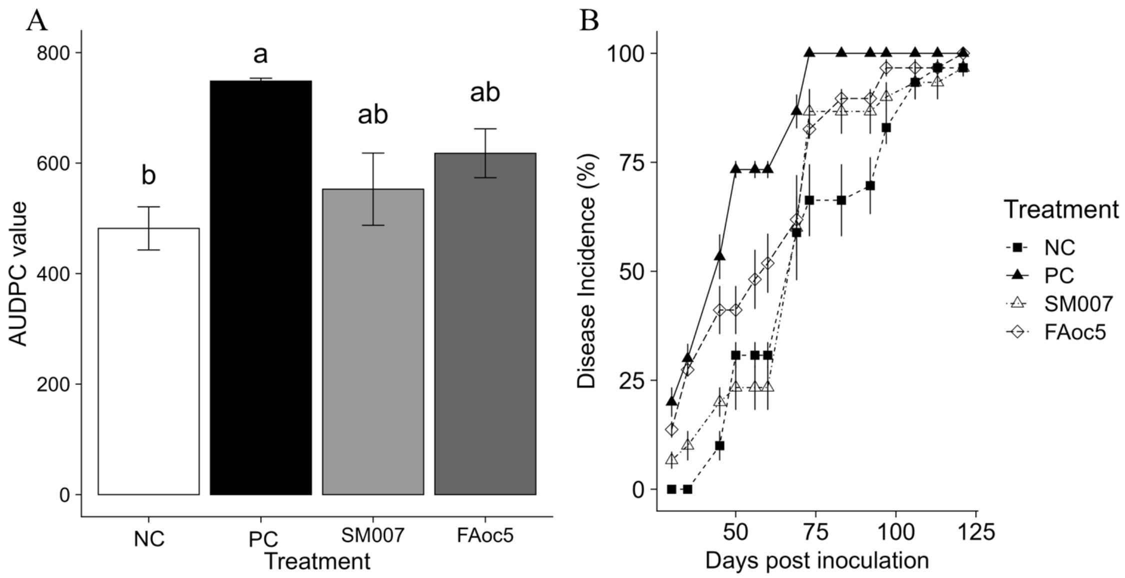

Fig. S1 Mature seedling experiments. (A) Area under disease progression curve (AUDPC) values and (B) Above-ground disease incidence in each plot. after cultivating mature plants in the Hokkaido University field following transplantation into pathogen-infested field soil and incorporation of wheat bran inoculated with each nonpathogenic strain. Different characters indicate significant differences ($P < 0.05$) based on Tukey-kramer's test for AUDPC values ($n = 3$).

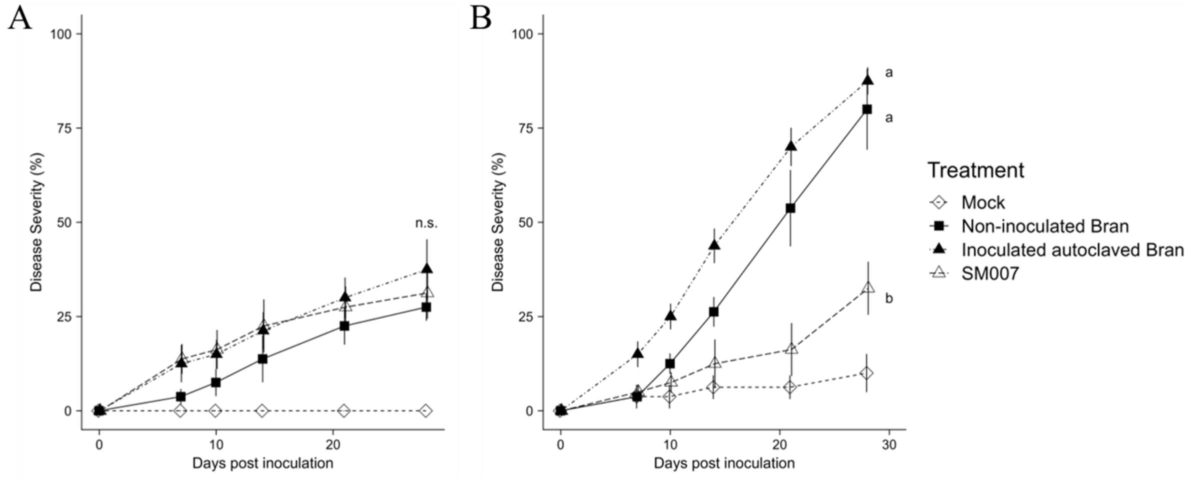

Fig. S2 Disease progression of Fusarium crown and root rot in asparagus seedlings after transplantation into pathogen-infested soil (1.0×10^4 cfu/g soil) following the application of treated wheat bran (10 g/pot). The graph was created using *ggplot* function in R. Each plot represents the mean disease severity for each treatment at each dpi. Results from two independent experiments are displayed separately due to differences in the density of nonpathogenic strain SM007 in wheat bran (5.4×10^4 cfu/g fresh bran and 8.2×10^5 cfu/g fresh bran, respectively). Aboveground disease severity was scored on a scale of 0 to 4, and calculated as Σ ([1A + 2B + 3C + 4D] /4 N) × 100, where A, B, C and D represent the number of plants rated 1, 2, 3, or 4, respectively and N is the total number of plants. Different characters indicate significant differences ($P < 0.05$) based on Tukey-kramer's test for AUDPC values ($n = 4$). The character "n.s." means no significance based on the Tukey-kramer's test ($P > 0.05$). In the second experiment, a few plants in the Mock treatment exhibited aboveground yellowing symptoms; however, disease incidence was evaluated as zero due to the absence of root lesions at the end of the experiment.

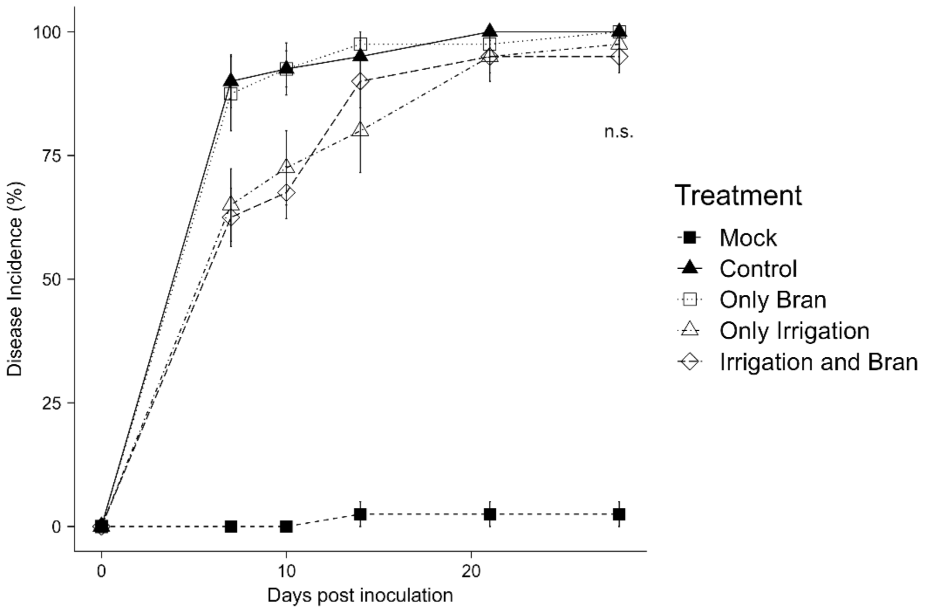

Fig. S3 Disease incidence of Fusarium crown and root rot following the transplantation of asparagus seedlings into pathogen-infested soil after inoculation with nonpathogenic strain SM007, either by irrigating bud-cell suspension or incorporating treated wheat bran. Five treatments were prepared, as follows: plants irrigated with Sterilized distilled water and transplanted into non-infested soil with IAB (Mock); the identical process but transplanted into infested soil (Control); plants irrigated with SDW and transplanted into infested soil with SM007-inoculated bran; plants irrigated with bud-cell suspension and transplanted into infested soil with IAB; and the identical process but using SM007-inoculated bran. Disease incidence and the area under the disease-progress curve were scored. Treatments were arranged in a randomized block design with four replicate pots per treatment, and the experiment was conducted twice (transplanting dates: October 31 and November 8, 2023). In both experiments, most plants exhibited severe wilt symptoms and died by 28 dpi. No treatments showed statistically significant differences compared to control. Each plot represents the mean disease incidence for each treatment at each dpi ($n = 8$).

Supplementary Tables

Table S1 Names and origins of the strains used as biocontrol agents in pre-screening experiment.

Session	Strain	Origin	Disease severity	Disease severity in control	Suppression value	Biocontrol effects[a]	Virulence to other plants
1	FAoc1	Asparagus	75	88	13		
1	FAoc2	Asparagus	83	88	5.0		
1	FAoc3	Asparagus	87	88	1		
1	FAoc4	Asparagus	82	88	6.7		
1	SM007	Soil	58	88	30	*	
2	FAoc5	Asparagus	38	68	30	*	
2	FAor1	Asparagus	57	68	11		
2	Mdc M-3	Strawberry	35	68	33	*	○[b]
2	Hok1	Wheat	50	68	18		
2	Mo4-62	Soil	58	68	10		
3	kam19	Wheat	48	73	25		
3	Mo4-40	Soil	50	73	23	*	
3	Mo4-31	Soil	31	73	41	*	
3	Mo4-52	Soil	42	73	31	*	
3	FAor2	Asparagus	38	73	35	*	
4	Mo4-47	Soil	33	65	32	*	
4	MAFF235727 (f. sp. *phaseoli*)	Common bean	58	65	7		○[c]
4	96-3k2 (f. sp. *adzukicola*)	Adzuki bean	42	65	23		○[d]
4	niho (f. sp. *cepae*)	Onion	38	65	27		○[e]

Severely diseased plants were those with root lesions exceeding 75%. Each strain was applied through root-dip inoculation using its respective bud-cell suspension before transplanting the seedlings into pots containing infested soil. After 2-4 weeks of cultivation, plants were up-rooted and disease severity was evaluated based on the root

lesion. Disease severity was scored on a scale of 0 to 4, and calculated as $\Sigma([1A + 2B + 3C + 4D] / 4\,N) \times 100$, where A, B, C and D represent the number of plants rated 1, 2, 3, or 4, respectively and N is the total number of plants.

The experiments were conducted across four sessions. Suppression value of each strain was calculated using the following formula:

suppression value = (mean of disease severity in control - mean of disease severity in biocontrol treatments).

[a] Strains with a suppression value of 30 or higher are marked with an asterisk.

[b] Virulent to *Brassicaceae* crops.

[c] Virulent to *Phaseolus vulgaris.*

[d] Virulent to *Vigna angularis.*

[e] Virulent to *Allium cepa.*

Table S2 Primers and PCR conditions following O'donnell *et al.* (2022)

	EF-1α	*RPB2*
Forward Primer	EF-1: ATGGGTAAGGARGACAAGAC	RPB2-5f2: GGGGWGAYCAGAAGAAGGC
Reverse Primer	EF-2: GGARGTACCAGTSATCATG	fRPB2-7cR: CCCATRGCTTGYTTRCCCAT
PCR conditions	94℃ for 3min, 35 cycles of 94℃ for 30 sec; 60℃ for 45 sec; 72℃ for 30 sec, and 72℃ for 7 min.	95℃ for 1 min, 30 cycles of 95℃ for 30 sec; 58℃ for 30 sec; 72℃ for 30 sec, and 72℃ for 10 min.

Table S3 Sequence data from ex-type strain used for phylogenetic analysis

Species	Strain	*EF1-α*	*RPB2*
Fusarium callistephi	CBS 187.53	MH484996	MH484875
Fusarium carminascens	CBS 144738	MH485028	MH484937
Fusarium commune	CBS 110090	AF362263	MW934368
Fusarium cugenangense	Indo203	LS479757	LS479308
Fusarium curvatum	CBS 238.94	MH484984	MH484893
Fusarium duoseptatum	Indo109	LS479688	LS479239
Fusarium elaeidis	CBS 217.49	MH484961	MH484870
Fusarium fabacearum	CBS 144743	MH485030	MH484939
Fusarium foetens	CBS 110286	MT011001	MW928825
Fusarium glycines	CBS 144746	MH485033	MH484942
Fusarium gossypinum	CBS 116613	MH485000	MH484909
Fusarium grosmichelii	Indo19	LS479744	LS479295
Fusarium nirenbergiae	CBS 840.88	MH484978	MH484887
Fusarium oxysporum	CBS 144134	MH485044	MH484953
Fusarium tardichlamydosporum	Indo158	LS479729	LS479280
Fusarium sp.	CBS 128.81	MH484975	MH484884
Fusarium sp.	CBS 680.89	MH484980	MH484889
Fusarium sp.	CBS 130323	MH485018	MH484927
